# Major Histocompatibility Complex class I proteins are critical for maintaining neuronal structural complexity in the aging brain

**DOI:** 10.1038/srep26199

**Published:** 2016-05-27

**Authors:** Maciej J. Lazarczyk, Julia E. Kemmler, Brett A. Eyford, Jennifer A. Short, Merina Varghese, Allison Sowa, Daniel R. Dickstein, Frank J. Yuk, Rishi Puri, Kaan E. Biron, Marcel Leist, Wilfred A. Jefferies, Dara L. Dickstein

**Affiliations:** 1 Fishberg Department of Neuroscience, Icahn School of Medicine at Mount Sinai, New York, NY 10029, USA; 2 Friedman Brain Institute, Icahn School of Medicine at Mount Sinai, New York, NY 10029, USA; 3 Department of Mental Health and Psychiatry, Division of General Psychiatry, University Hospitals of Geneva, Faculty of Medicine of the University of Geneva, Geneva, Switzerland; 4 University of Konstanz, Doerenkamp-Zbinden, Universitätsstrasse. 10, 78457 Konstanz, Germany; 5 Michael Smith Laboratories, The University of British Columbia, 2185 East Mall, Vancouver, British Columbia, V6T 1Z4, Canada; 6 Department of Microbiology and Immunology, University of British Columbia, 1365-2350 Health Sciences Mall, Vancouver, BC, V6T 1Z3, Canada; 7 Centre for Blood Research, University of British Columbia, 2350 Health Sciences Mall, Vancouver, BC, V6T 1Z3, Canada; 8 Department of Zoology, University of British Columbia, 2370-6270 University Blvd., Vancouver, BC, V6T 1Z4, Canada; 9 Department of Medical Genetics, 1364-2350 Health Sciences Mall, Vancouver, BC, V6T 1Z3, Canada

## Abstract

Major histocompatibility complex class I (MHCI) proteins have been implicated in neuronal function through the modulation of neuritogenesis, synaptogenesis, synaptic plasticity, and memory consolidation during development. However, the involvement of MHCI in the aged brain is unclear. Here we demonstrate that MHCI deficiency results in significant dendritic atrophy along with an increase in thin dendritic spines and a reduction in stubby spines in the hippocampus of aged (12 month old) mice. Ultrastructural analyses revealed a decrease in spine head diameter and post synaptic density (PSD) area, as well as an increase in overall synapse density, and non-perforated, small spines. Interestingly, we found that the changes in synapse density and morphology appear relatively late (after the age of 6 months). Finally, we found a significant age dependent increase in the levels of the glutamate receptor, GluN2B in aged MHCI knockout mice, with no change in GluA2/3, VGluT1, PSD95 or synaptophysin. These results indicate that MHCI may be also be involved in maintaining brain integrity at post-developmental stages notably in the modulation of neuronal and spine morphology and synaptic function during non-pathological aging which could have significant implications for cognitive function.

There is increasing evidence for the role of the major histocompatibility complex class I (MHCI), a protein complex best known for antigen presentation and immunological surveillance in the adaptive immune system, in a second function within the central nervous system (CNS). Originally, the brain was considered to be “immunologically privileged”, with low expression of MHCI unless evoked in response to traumatic injury or functional impairment in learning and memory^1^,^2^,^3^. However, it has now been demonstrated that MHCI is expressed on neurons during development and early adulthood in brain regions including the neocortex^4^, hippocampus^5^,^6^, spinal motoneurons^7^, and *substantia nigra*^8^. In the developing CNS, MHCI has been shown to modulate synaptic plasticity, axonal and dendritic morphogenesis, and neuronal polarity^6^,^9^,^10^,^11^,^12^,^13^; all functions that are completely distinct from its role in the peripheral immune system.

Little is known about MHCI function in the aging brain. During healthy aging, there are substantial changes in neuronal complexity, structural reorganization of dendritic spines, and disturbances in synaptic signaling in pyramidal neurons located in the prefrontal, superior temporal, and precentral cortex, and hippocampus (reviewed in^13^). These changes are thought to underlie age-related impairments in learning and memory that may occur during healthy aging. However, the molecular mechanisms that account for these age-related structural and synaptic alterations have not been fully illuminated. In rat hippocampus, increased neuronal expression of MHCI and other functionally related proteins, such as β2-microglobulin (β2M), transporter associated with antigen processing (TAP), paired immunoglobulin-like receptor B (PirB), and killer cell lectin-like receptor (Klra; also known as Ly49) was found in cognitively impaired as well as cognitively intact aged rats compared to adult rats^14^. In contrast, MHCI expression in humans appears to be significantly increased in cognitively intact oldest-old (≥87 years of age) individuals and decreased in cognitively impaired oldest-old, relative to younger-old (≤86 years of age) cognitively intact individuals^15^. Moreover, recent genome association studies found alleles of human leukocyte antigen-A, one of the three human MHCI genes, associated with increased risk of Alzheimer’s disease^16^,^17^. These data suggest that, depending on the species and the cognitive tasks assessed, an age-related increase in MHCI is important in regulating and preserving cognitive function and thus may be a crucial mechanism for maintaining memory function associated with successful aging. As variability in neuronal and spine morphology has been associated with memory formation and cognitive function, MHCI may regulate memory and cognition through the formation and/or elimination of synapses, similar to its function during development^18^. MHCI is known to modulate excitatory glutamate receptor function^11^,^19^,^20^,^21^,^22^. Altered activity of these receptors has been linked to dendritic spine clustering^20^ and their expression at the synapse correlated with age-related cognitive decline^19^,^21^.

While evidence for a role of MHCI in the CNS is quite compelling, much of the existing data have been generated in transgenic mouse models lacking TAP1, β2M or a combination of the two. The TAP1 protein is essential for transporting cytoplasmic peptides into the endoplasmic reticulum where they may be loaded onto MHCI, while β2M forms the light chain component of MHCI and is necessary for the proper folding, protein stability and export of MHCI to the cell surface. Mice that are either TAP1^−/−^ or β2M^−/−^ have a markedly reduced presence of MHCI at the cell surface^3^,^4^,^23^,^24^. However, MHCI is detectable at the surface of cells from the TAP1^−/−^ and β2M^−/−^ mice under certain conditions^25^, though it is undetectable in combined double knockouts^23^,^26^. It is also possible that knocking out these proteins can result in phenotypes related to their other functions, *e.g*. the association of β2M with the human hemochromatosis protein and iron transport. In contrast, knockout of the H2-K and H2-D genes in the K^b^D^b−/−^ mice results in loss of the heavy chain of MHCI and thereby complete absence of MHCI.

To better understand the role of MHCI in the maintenance of synaptic plasticity in adults in a brain area implicated in learning and memory, we examined neuronal complexity, spine and synapse density and morphology in hippocampal neurons from 12 month old (aged) K^b^D^b−/−^, TAP^−/−^, and β2M^−/−^ mice, as well as younger animals. We also examined expression levels of post-synaptic proteins, post synaptic density protein (PSD95), α-amino-3-hydroxy-5-methyl-4-isoxazolepropionic acid (AMPA)- and *N*-Methyl-D-aspartate (NMDA)-type glutamate receptors, and pre-synaptic proteins, the major synaptic vesicular protein, synaptophysin and vesicular glutamate transporter 1 (VGluT1). Our experiments demonstrate that aged MHCI double knockout (K^b^D^b−/−^) mice present with significant neuronal atrophy in the CA1 region of the hippocampus, while β2M^−/−^ and TAP^−/−^ mice, with partial loss of MHCI, showed less obvious or no changes in neuronal complexity. Aged K^b^D^b−/−^ mice also had alterations in spine and synaptic morphology and density, and increased expression of the GluN2B NMDA receptor compared to both wild-type (WT) controls and young K^b^D^b−/−^ mice. These changes appeared in post-developmental stages, after the age of 6 months. These studies suggest that MHCI plays a role in the maintenance of neuronal integrity upon aging and may be essential for normal hippocampal-dependent memory in the aging brain.

## Results

### Absence of MHCI does not affect laminar cell layers

**Figure 1:**
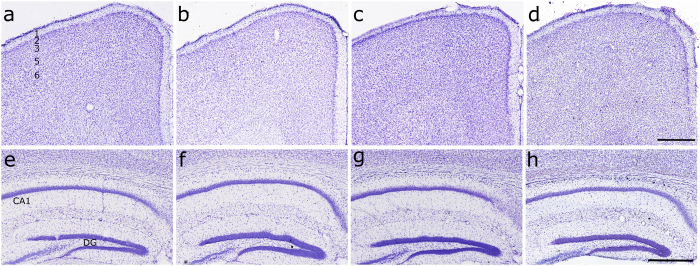
Loss of MHCI has no effect on cytoarchitectural stratification. Nissl-stained coronal sections (50 μm) of adult brains from control (**a,e**), K^b^D^b−/−^ (**b,f**), β2M^−/−^ (**c,g**), and TAP^−/−^ (**d,h**) mice at the prefrontal and hippocampal levels along the rostrocaudal axis of the neocortex. Layers of the neocortex are represented in Panel **a** (1,2,3,5,6); Abbreviations: CA1, cornus ammonis1; DG, dentate gyrus. Scale bar = 500 μm.

Proper neocortex function depends on the production and positioning of a correct number of excitatory and inhibitory neurons into distinct cellular layers. To investigate whether MHCI has an effect on brain architecture and lamination, MHCI deficient K^b^D^b−/−^, β2M^−/−^ and TAP^−/−^ mice were compared to WT at 12 months of age by Nissl staining (Fig. 1). Examination of the brains of mice from all genotypes showed no abnormalities in the organization of cortical neurons with a process-rich layer I at the pial surface followed by appropriately organized neuronal layers 2–6 (Fig. 1a–d). The cytoarchitecture in the hippocampi of MHCI deficient mice also appeared normal when compared to WT mice (Fig. 1e–h).

### MHCI deficiency alters apical dendritic morphology

**Figure 2:**
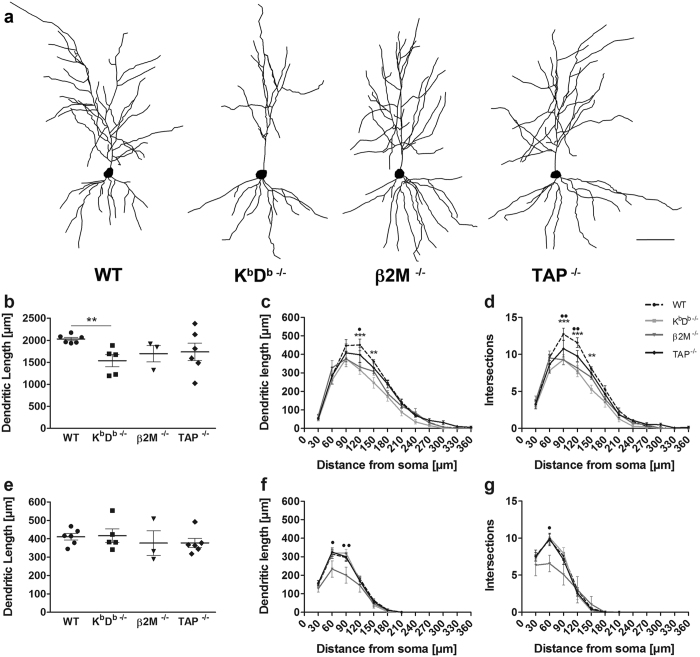
Loss of MHCI results in attrition of apical dendritic length and significant reduction in neuronal complexity in aged mice. (**a**) Representative cell tracings from each genotype. Scale bar = 50 μm. (**b**) Analysis of apical dendritic length. (**c,d**) Sholl analyses of apical dendrites. (**e**) Analysis of basal dendritic length. (**f,g**) Sholl analyses of basal dendrites. **p < 0.01, ***p < 0.001, K^b^D^b−/−^ vs. WT. ^•^p < 0.05, ^••^p < 0.01, β2M^−/−^ vs. WT. Data represent means ± SEM.

We assessed whether CA1 neurons from 12 month old MHCI deficient K^b^D^b−/−^, β2M^−/−^ and TAP^−/−^ mice would display differences in dendritic length and complexity compared to neurons from WT mice. A total of 18 animals and approximately 5 neurons per animal met the inclusion criteria for use in neuronal reconstructions described in Materials and Methods (Table 1). Representative examples of CA1 dendritic arbor reconstructions of K^b^D^b−/−^, β2M^−/−^, TAP^−/−^, and WT neurons are depicted in Fig. 2a. Our results showed that K^b^D^b−/−^ mice had significantly shorter apical dendrites compared to WT (F_(1,9)_ = 10.76, *p* < 0.005). Similar effects were observed in the β2M^−/−^ and TAP^−/−^ mice; however, these trends did not reach significance (Fig. 2b). We then performed a Sholl analysis to identify any further changes in morphological complexity that may occur in the absence of MHCI. When comparing the numbers of intersections, we found a significant main effect of genotype (F_(3,176)_ = 4.206, *p* < 0.0226) and distance from soma (F_(11,176)_ = 277.6, *p* < 0.0001). Furthermore, apparent differences were also observed in dendritic length at varying distances from the soma (F_(11,176)_ = 258.3, *p* < 0.0001) and a trend for genotype effect was found (F_(3,176)_ = 2.515, *p* = 0.095). Specifically, we found significant decreases in dendritic length at 120 μm and 150 μm away from the cell body (Fig. 2c) as well as significant differences in total number of intersections at distances of 90 μm to 150 μm away from the cell body in K^b^D^b−/−^ compared to WT (Fig. 2d). Analysis of β2M^−/−^ mice showed significant decrease in dendritic length at 120 μm from the soma (Fig. 2c) and significantly reduced complexity at distances of 90 μm and 120 μm from the soma compared to WT (Fig. 2d). Comparisons between TAP^−/−^ and the WT showed no difference regarding the dendritic length or intersections.

Examination of the mean basal dendritic length revealed no main effect of MHCI on dendritic length in any of the mouse models (Fig. 2e). However, Sholl analyses of basal intersections showed significant effects of distance from soma (F_(6,96)_ = 266, *p* < 0.0001), but no effect of genotype on the number of intersections (F_(3,96)_ = 0.8866, *p* = 0.469). Sholl analysis of dendritic length also demonstrated significant difference with regards to distance from soma (F_(6,96)_ = 313.5, *p* = < 0.0001), but not with genotype (F_(3,96)_ = 1.710, *p* = 0.205). Specifically, β2M^−/−^ mice exhibited a significant decrease in basal dendritic length at 60 μm and 90 μm away from the soma and in the number of intersections at 60 μm from the soma compared to controls (Fig. 2f,g).

### Loss of MHCI alters dendritic spines and synapses

**Figure 3:**
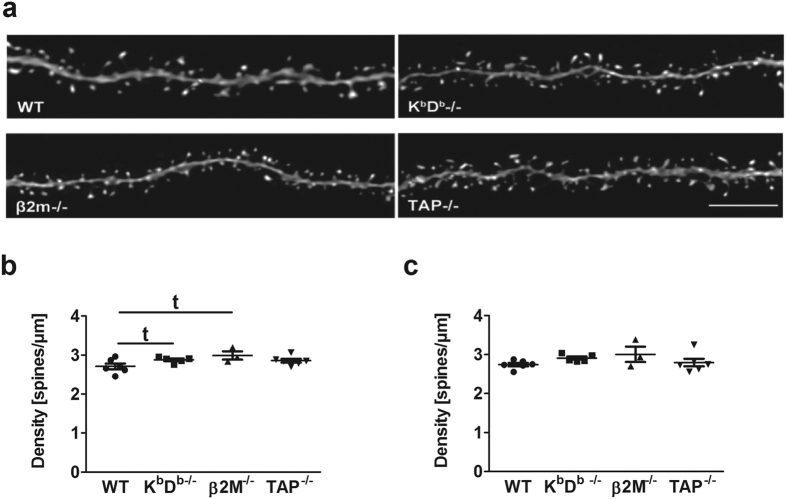
Absence of MHCI has no effect on overall spine density in aged mice. (**a**) Representative 3D renderings of apical dendritic segments of CA1 pyramidal neurons from WT, K^b^D^b−/−^, β2M^−/−^, and TAP^−/−^ mice. Scale bar = 5 μm. (**b,c**) Analysis of overall spine density in both apical and basal spines respectively. t indicates a trend towards significance as compared to WT. Data represent means ± SEM.

**Figure 4:**
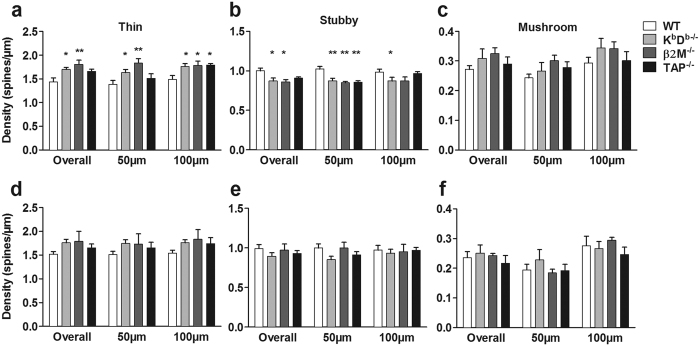
Absence of MHCI results in changes in spine density on CA1 apical dendrites in aged mice. There was a significant increase in the density of thin spines (**a**) and a decrease in stubby spines (**b**) on apical dendrites in K^b^D^b−/−^ and β2M^−/−^, but not TAP^−/−^, compared to WT, with no change in the density of mushroom spines (**c**). When the apical spine density was analyzed by distance from soma, increased spine density in K^b^D^b−/−^ and β2M^−/−^, but not TAP^−/−^, compared to WT was observed at 50 μm from the soma for thin spines (**a**), while all three knockout strains had fewer stubby spines (**b**) at this distance. At 100 μm from the soma, all three knockout strains had more thin spines (**a**) than the WT, while only K^b^D^b−/−^ had fewer stubby spines (**b**) compared to WT. MHCI loss had no effect on basal spine type across all genotypes (**d–f**). Note the different scales in the Y-axis for each spine type. (*p < 0.05, **p < 0.01 compared to WT). Data represent means ± SEM.

**Figure 5:**
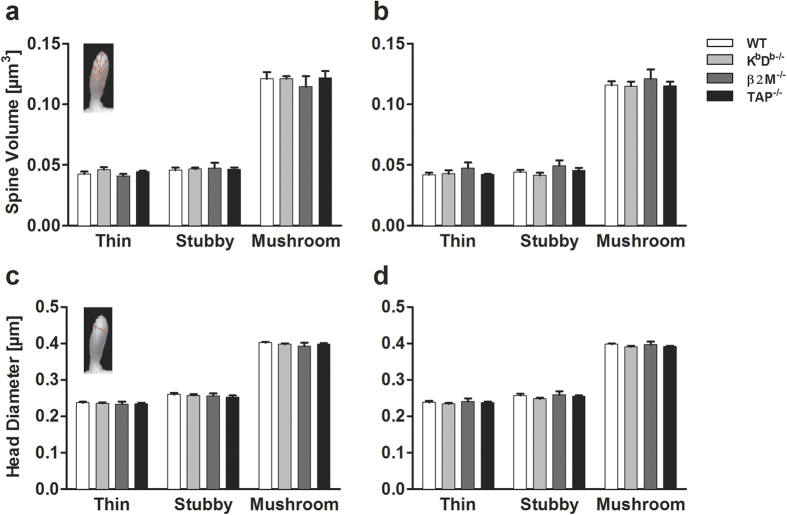
Absence of MHCI has no effect on spine head volume or diameter. Analysis of spine head volume and head diameter in both apical (**a,c**) and basal (**b,d**) spines revealed no significant differences in any of the MHCI knockout mice as compared to WT. Insets depict how NeuronStudio quantifies spine head volume and diameter. Data represent means ± SEM.

In order to assess the effect of MHCI on dendritic spine properties, such as changes in spine density, type, volume and head diameter, we analyzed a total of 78,361 spines from both apical and basal dendrites (3 dendritic segments/apical or basal dendrite/neuron and 5 neurons/animal; Fig. 3a; Table 1) in 12 month old mice. Figure 3a shows representative examples of apical dendritic segments of each genotype that were used to assess spine morphology. We found no difference in overall apical and basal spine density in all genotypes compared to WT (Fig. 3b,c). Spine morphology is known to influence spine function, and changes in spine size can play a substantial role in mediating cognitive function^27^,^28^. Therefore, we assessed whether the absence of MHCI influences the spine shape. Our analysis revealed significant increases in the overall density of thin spines (F_(3,16)_ = 5.014, p = 0.012) along with significant decreases in the density of stubby spines (F_(3,16)_ = 4.577, p = 0.017) on apical dendritic segments of all mouse groups compared to WT (Fig. 4a–c). Next, we analyzed spine type as a function of distance from the soma. Repeated-measures ANOVAs revealed in MHCI deficient animals a significant overall increase in thin spines (F_(3,16)_ = 4.964, p = 0.013) as well as with distance from soma (F_(1,16)_ = 8.829, p = 0.009). In particular, we found K^b^D^b−/−^ and β2M^−/−^ mice had more thin spines at a distance of 50 μm whereas K^b^D^b−/−^, β2M^−/−^ and TAP^−/−^ mice had more thin spines at 100 μm compared to WT. In contrast, there was a decrease in stubby spines in all of the genotypes compared to WT (F_(3,16)_ = 4.373, p = 0.015) at 50 μm from the soma, but not at 100 μm. There were no differences observed in mushroom spines. In regards to basal dendritic spines, we found no effect of MHCI deficiency in overall spine density of any spine type and in spines at distances of 50 μm and 100 μm from the soma (Fig. 4d–f). Finally, we examined whether MHCI had an effect on spine volume and head diameter (Fig. 5). We did not find any significant alterations in volume or head diameter in both apical and basal dendrites.

**Figure 6:**
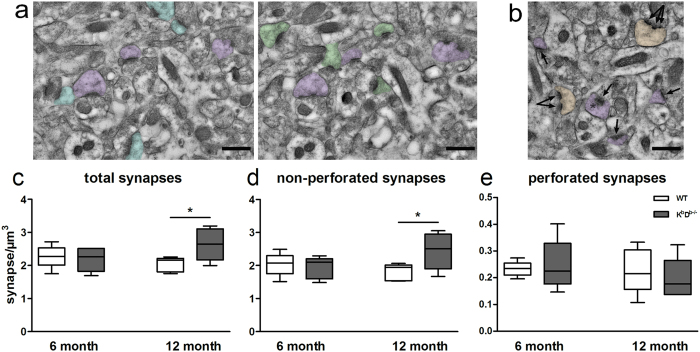
MHCI deficient mice exhibit an increase in synapse density. (**a**) Electron micrographs depicting synapses counted using the disector method. Only asymmetric axospinous synapses that were present in the reference panel (purple or blue), but not in the look-up panel were counted. Synapses present in both panels (green) are not included. (**b**) Non-perforated synapses (purple) are distingished from perforated synapses (orange) by the presence of a discontinuity within the PSD (arrows). Scale bars = 500 nm. No differences in (**c**) total, (**d**) perforated, (**e**) non-perforated synapse density were observed at 6 months of age however, aged (12 month old) MHCI deficient mice exhibit an increase in overall synapse density (**c**) and in non-perforated synapse density (**d**) with no changes in perforated synapse density (**e**). *p < 0.05 K^b^D^b−/−^ vs WT. Data represent means ± SEM.

**Figure 7:**
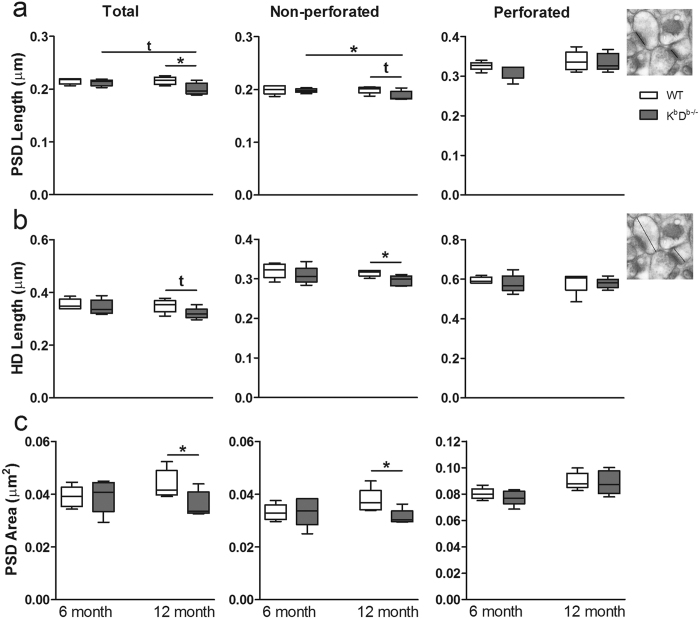
MHCI deficient mice exhibit alterations in synaptic structure with age. Quantification of synaptic ultrastructure of (**a**) PSD length (**b**) synapse HD, and (**c**) PSD area for overall, non-perforated, and perforated synapses in 6 and 12 month old K^b^D^b−/−^ mice compared to WT. Insets depict EM micrographs illustrating how PSD length and HD were quantified. *p < 0.05. t indicates a trend towards significance as compared to the indicated group. Data represent means ± SEM.

Given that MHCI deficiency in these 12 month old animals has an effect on spine density and can differentially affect spine morphology in different spine types, we chose to examine the ultrastructural changes resulting at the synaptic level due to MHCI deficiency. Since the largest effect was seen in K^b^D^b−/−^ mice, we only used this MHCI-deficient mouse model for ultrastructural analysis. To determine if MHCI affects synapse density and the formation of perforated and non-perforated synapses (Fig. 6a,b), we examined synapses on spines from the stratum radiatum (SR) dendritic domain of the hippocampal CA1, which encompasses the proximal regions of the apical dendrites where we observed the most prominent changes in spines. Approximately 4700 synapses were reconstructed (477/animal on average) across serial electron microscopy (EM) sections using the disector method. Analysis of the density of synapses from SR revealed a significant increase in synapse density in aged K^b^D^b−/−^ compared to WT mice (*t*_(8)_ = 2.388, p = 0.036; Fig. 6c). Further analysis of synapse type revealed a significant increase in density of non-perforated synapses in K^b^D^b−/−^ mice (*t*_(8)_ = 2.235, p = 0.049; Fig. 6d) and no difference in the density of perforated synapses in K^b^D^b−/−^ compared to WT mice (Fig. 6e). We next examined synaptic head diameter (HD), post synaptic density (PSD) lengths and total PSD area of synapses across K^b^D^b−/−^ and WT animals. The data showed a significant decrease in overall PSD length and area with a slight decrease in total HD in K^b^D^b−/−^ mice as compared to WT (*t*_(8)_ = 2.597, p = 0.03; *t*_(8)_ = 2.385, p = 0.04; *t*_(8)_ = 2.003, p = 0.08 respectively; Fig. 7). Interestingly, separate analysis of non-perforated spines showed a decrease in the same structural parameters in K^b^D^b−/−^ mice, with significant changes in HD and PSD area and a trend in PSD length (*t*_(8)_ = 2.753, p = 0.02; *t*_(8)_ = 2.602, p = 0.03; *t*_(8)_ = 2.233, p = 0.056 respectively). No differences were observed in these parameters for perforated synapses.

In order to verify whether this phenotype appears early and persists or emerges only upon aging, we replicated the analysis of synapse density and morphology in 6 month old WT and K^b^D^b−/−^ mice (young adult) and compared those findings to the 12 month old mice (aged). For 6 month old mice, approximately 4500 synapses were reconstructed (454/animal on average) across serial EM sections using the disector method. We found no differences in synapse density (perforated or non-perforated) in 6 month old MHCI deficient K^b^D^b−/−^ mice compared to WT (Fig. 6). When we assessed PSD length, HD, and PSD area in the younger animals, we also did not observe any ultrastructural differences in K^b^D^b−/−^ compared to WT (Fig. 7). Comparison of 6 month and 12 month old K^b^D^b−/−^ mice showed an increase in overall synapse density and an increase in non-perforated synapse density; however, these did not reach significance (Fig. 6c–e). Further analysis showed a decrease in total PSD length and a significant decrease in non-perforated synapse PSD length (*t*_(8)_ = 2.101, p = 0.07; *t*_(8)_ = 2.313, p = 0.04 respectively; Fig. 7a). The increased density and decreased size of non-perforated synapses in older mice lacking MHCI could significantly impact synaptic receptor distribution at the synapse as well as synaptic function in aging.

### MHCI deficiency results in an increase in NMDA receptor expression

**Figure 8:**
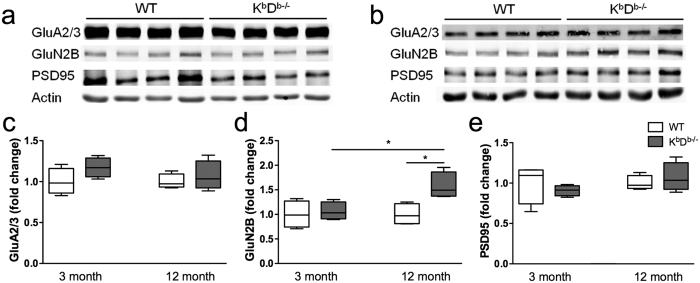
NMDA receptor expression is increased in MHCI deficient mice. Representative Western blots of hippocampal homogenates and analysis of integrated fluorescent intensities of GluA2/3, GluN2B, and PSD95 in (**a**) 3 month and (**b**) 12 month old K^b^D^b−/−^ and WT mice. The representative blots were cropped for concise presentation and the respective full-sized blots are presented in Supplementary Fig. 1. There was a significant increase in GluN2B (**d**) in 12 month old K^b^D^b−/−^ mice compared to WT mice of the same age or to 3 month old K^b^D^b−/−^ mice. There was no difference in GluA2/3 (**c**) and PSD95 (**e**) expression in all ages and genotypes. All proteins were normalized to actin. Histograms represent means ± SEM. *P < 0.05.

**Figure 9:**
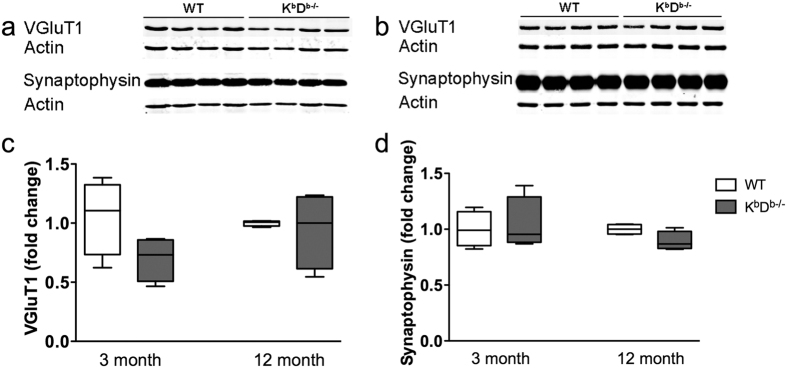
Presynaptic markers are unchanged in MHCI deficient mice. Representative Western blots of hippocampal homogenates and analysis of integrated fluorescent intensities of VGluT1 and synaptophysin in (**a**) 3 month and (**b**) 12 month old K^b^D^b−/−^ and WT mice. The representative blots were cropped for concise presentation and the respective full-length blots are presented in Supplementary Fig. 2. There was no significant change in VGluT1 (**c**) or synaptophysin (**d**) in 3 or 12 month old K^b^D^b−/−^ mice compared to WT mice of the same age. All proteins were normalized to actin. Histograms represent means ± SEM.

Next, we examined whether the MHCI deficient mice exhibited alterations in synaptic protein expression. We analyzed levels of postsynaptic scaffolding protein, PSD95, the NMDA receptor, GluN2B, the α-AMPA subunits, GluA2/3 (Fig. 8a,b; Supplementary Fig. 1), and presynaptic proteins, VGluT1 and synaptophysin, in the hippocampus of 3 and 12 month old K^b^D^b−/−^ and WT mice (Fig. 9a,b; Supplementary Fig. 2). We did not see any difference in the expression of GluA2/3, PSD95, VGluT1 or synaptophysin in the hippocampus of K^b^D^b−/−^ and WT mice at either age (Figs 8c,e and 9). We did note a significant increase in GluN2B expression in K^b^D^b−/−^ mice in comparison to WT littermates in the 12 month old (*t*_(6)_ = 3.312, p = 0.02 Fig. 8d), but not 3 month old animals. GluN2B expression was also increased in 12 month old K^b^D^b−/−^ as compared to 3 month old animals of the same genotype (*t*_(6)_ = 3.313, p = 0.02 Fig. 8d).

## Discussion

Our results highlight that the complete absence of MHCI results in decreased apical dendritic complexity in CA1 pyramidal neurons in aged mice. Moreover, the loss of MHCI resulted in a shift of dendritic spine type with an increase in thin spines and a decrease in stubby spines. These changes appear to occur with advanced age, as in contrast to 12 month old animals, 6 month old mice did not have significant changes in synapse density, and synapse morphology compared to controls. We also show that the expression of the GluN2B NMDA receptor is increased in aged but not young MHCI-deficient mice, while PSD95, GluA2/3, VGluT1 and synaptophysin levels are unchanged at both ages. Taken together, these data provide evidence for a functional role of MHCI in the healthy aging brain in maintaining synaptic plasticity in the hippocampus.

Previous studies in the developing brain have demonstrated a role for MHCI in neurite outgrowth. For example, cultured embryonic hippocampal neurons from K^b^D^b−/−^, but not β2M^−/−^, mice showed slower neuritogenesis and neurite outgrowth^28^. Here we show that MHCI is also necessary for the maintenance of dendritic complexity in the aged brain as K^b^D^b−/−^ mice exhibited a significant decrease in neuronal apical dendritic length and complexity in the hippocampal CA1 compared to WT. Other studies in adult mice corroborate our finding that MHCI has an effect on maintaining neuronal complexity. A recent study by Adelson *et al*.^29^ showed that basal dendrites are atrophied in the visual cortex of K^b^D^b−/−^ mice^29^. In addition, deficits of MHCI do not appear to affect neurogenesis in the adult mouse brain, as demonstrated in TAP^−/−^ mice^30^. Based on these studies and the fact that increased MHCI expression is found in oldest-old non-demented individuals^15^, it is plausible that MHCI has a crucial function in maintaining neuronal integrity in healthy aging. Interestingly, over-expression of the H2-D^b^ heavy chain of MHCI may be either beneficial or deleterious during development, with one study in cultured embryonic hippocampal neurons reporting faster neurite outgrowth^28^ and another study showing fewer neurons in the CA1 layer of the mouse hippocampus following H2-D^b^ upregulation^31^. While the data is conflicting, it is apparent that MHCI is essential for the establishment and maintenance of neurite outgrowth and complexity during both development and aging, and that any change in the dynamic homeostasis of MHCI expression can have significant and different effects based on the age at which such a change occurs. Alterations in the levels of MHCI may render neurons unable to sustain the dendritic complexity required for acquiring and sustaining neuronal connections during aging.

MHCI has also been implicated as a key player in synaptogenesis and synaptic pruning. Research has shown that β2M^−/−^, TAP^−/−^, and MHCI deficient mice all lack efficient or appropriate synaptic pruning during development^6^ while over-expression of the H2-K^b^ heavy chain of MHCI in cultured neurons results in a decrease in synapse density of both excitatory GluN2A/B and inhibitory γ-aminobutyric acid synapses^32^. Moreover, it appears that the H2-K^b^ and H2-D^b^ alleles are required for synapse elimination in CNS neurons, in particular in the retinogeniculate system during development^33^, with increased spine density on basal dendrites of visual cortex neurons in K^b^D^b−/−^ mice^29^. In the hippocampus, altered synapse density is observed, specifically an increase in CA3 and no changes in CA1 in β2M^−/−^TAP^−/−^ mice and in K^b^D^b−/−^ mice at postnatal day 30–32^34^. Our results are complementary to these data as we did not observe any differences in CA1 spine density at 6 months of age. However, we did observe an increase in the density of thin spines, overall synapse density, and an increase in non-perforated synapses in 12 month old MHCI deficient K^b^D^b−/−^ mice compared to WT. Taken together with the lack of age-related changes in synapse density between 6 and 12 month old WT mice, these data likely indicate a change that occurs during late adulthood and argues for the involvement of MHCI in synapse maintenance upon aging. The increased thin spines in the hippocampus of aged MHCI deficient mice may also undergo synapse reformation cycles, indicating weaker or less persistent synaptic connections. Weakening of synapses has been shown to play a critical role in a homeostatic mechanism to improve neural connectivity^35^, and we believe that if the aged MHCI-deficient mice do in fact lack the ability to eliminate synapses, they will not have the ability to promote the emergence of new circuits with reinforced connections. Alternatively, since there is a close association between synaptic transmission and dendritic arbor development in neurons^36^, an increase in thin spines may compensate for a lack of excitatory input as a result of the observed decrease in dendritic complexity. The mechanism by which MHCI exerts its effects on neuronal plasticity in the aged hippocampus, and whether these effects are a result of signals or input from other areas in the brain or the periphery, remains to be determined.

Given that we observed an increase in thin spines and the decrease in PSD length and area in K^b^D^b−/−^ mice, we sought to examine whether proteins involved in excitatory neurotransmitters were affected. MHCI has been previously linked to altered excitatory neurotransmitter function. Evidence from β2M^−/−^TAP^−/−^ mice show increased NMDAR-mediated long-term potentiation^11^, with no changes in the levels or distribution of the NMDA receptor subunits^20^. However, NMDA treatment increased cell-surface AMPA receptor trafficking in neurons from these mice. Our results indicate an increase in GluN2B expression in 12 month K^b^D^b−/−^ mice as compared to WT, with no change in the GluA2/3 expression. The importance of the GluN2B receptor in modulating spine density and thereby memory has been demonstrated by selective genetic ablation of this receptor in the CA1, which resulted in decreased apical spine density and impaired corticohippocampal learning and memory^37^. This receptor subunit has been shown to affect spine motility and formation during development^38^ as well as memory function in aging^39^ and our results imply it may play a key role in CA1 dendrite and spine maintenance in the context of MHCI deficiency in the aged brain. Whether the observed upregulation of GluN2B is a direct compensatory mechanism or a consequence of synaptic changes induced by loss of MHCI remains to be resolved.

In summary, we provide evidence that the presence of MHCI significantly affects processes pertaining to synaptic maintenance and function, including neuronal dendritic length and complexity, spine density and morphology, and excitatory receptor expression in aging. Previous studies have shown that MHCI has an effect on hippocampal-dependent memory function as shown by the deletion of both β2M^−/−^ and TAP^−/−^ 11. The decreased dendritic arborization in CA1 neurons, together with increased thin spines and increased GluN2B receptor expression support this observation that complete deficiency of MHCI, plays a role in memory formation. Understanding the role of MHCI in synaptic formation and plasticity during the non-pathological aging process remains a vital issue in understanding the function of MHCI in the CNS and may provide new targets for treatments that maintain synaptic connectivity associated with memory and learning with age and in degenerative diseases.

## Materials and Methods

### Experimental animals

MHCI deficient mice (H-2K^b^ H-2D^b^ double knockout, K^b^D^b−/−^)^40^ were a gift from Dr. John W. Chamberlain (The Hospital for Sick Children, Toronto, Canada). K^b^D^b−/−^ mice, on a C57BL/6 background, lack the H2-K^b^ and H2-D^b^ MHCI genes and as such express no detectable MHCI-region associated heavy chains and cannot elicit an immune response^40^. Many other studies investigating MHCI in the CNS use mouse knockouts of β2M, TAP1 or both. β2M is important given that functional MHCI is a trimer consisting of the heavy chain, β2M, and an antigenic peptide generated from proteosomal degradation^7^. In order for heavy chain expression and antigen presentation to occur, β2M and peptide must be present^24^,^41^. In their absence, both surface and intracellular levels of MHCI are down-regulated. β2M deficient mice (β2M^−/−^; The Jackson Laboratory Bar Harbor USA) have little to no expression of MHCI on the cell surface. The TAP1 and 2 proteins are essential for the transport of the functional MHCI unit to the cell surface. Deletion of the TAP1 protein results in little to no MHCI at the cell surface. TAP1 deficient mice (TAP^−/−^; the Jackson Laboratory) are defective in stable assembly and intracellular transport of MHCI molecules and show significantly reduced levels of MHCI at the cell surface. Both mouse lines display altered synaptic plasticity in the hippocampus and abnormal patterning of visual system connections^5^,^6^. Therefore, brains of mice deficient in β2M and TAP1 were studied here as MHCI “loss of function” in addition to the K^b^D^b−/−^ mice where there is a complete knock-out of MHCI. C57BL/6 (WT) mice were used as controls. All mice were maintained at the University of British Columbia, Vancouver, BC, Canada. For neuron and spine morphological analyses, 6 and 12 month old animals were used. For biochemistry, 3 and 12 month old animals were used. Mice were kept under a 12-hour light/dark cycle and fed standard lab chow and water *ad libitum*. All animal experiments were performed at the University of British Columbia, Vancouver, BC, Canada and were conducted in compliance with the approved protocols. All protocols were approved by the University of British Columbia Animal Care Committee under the direction of the Canadian Council for Animal Care.

### Perfusion and tissue processing

Mice were anesthetized with Avertin (250–500 mg/kg, intraperitoneal) and transcardially perfused with 1% paraformaldehyde (PFA) in phosphate buffered saline (PBS; pH 7.4) followed by 4% PFA/0.125% glutaraldehyde in PBS as described previously^42^,^43^,^44^. The brains were removed, hemisected, and postfixed overnight at 4 °C in 4% PFA/0.125% glutaraldehyde in PBS. The hemispheres were then sectioned on a Vibratome (Leica VT1000S) into 200 μm-thick sections for cell loading experiments and 250 μm for EM. All sections were stored at 4 °C in PBS + 0.01% azide until ready for use. In addition, 50 μm sections were also obtained from each animal and stained for Nissl (0.5% cresyl violet in 0.3% acetic acid) to assess cytoarchitecture.

### Intracellular dye Injections

For intracellular injections, sections were incubated in 4′,6-diamidino-2-phenylindole (DAPI) for 5 minutes to reveal the cytoarchitectural features of the pyramidal layer of CA1 of the hippocampus. The sections were then mounted on nitrocellulose and immersed in PBS. Neurons were impaled with a sharp glass micropipette and loaded with 5% Lucifer Yellow in distilled water under a direct current of between 3 and 8 nA for 5–10 minutes, or until dye had completely filled distal processes and no further loading was observed^42^,^43^,^44^. Five to ten neurons were loaded per animal and spaced far enough apart from each other to prevent overlapping of dendrites. The sections were then mounted on gelatin-coated glass slides and cover slipped in Fluoromount G mounting media (Beckman Coulter).

### Neuron and dendritic reconstruction

To be included in the analysis, a loaded neuron had to satisfy the following criteria: (1) reside within the pyramidal layer of the CA1 as defined by cytoarchitectural characteristics; (2) demonstrate complete filling of dendritic tree, as evidenced by well-defined endings; and (3) demonstrate intact tertiary branches, with the exception of branches that extended beyond 50 μm in radial distance from the cell soma^42^,^43^,^44^. Neurons meeting these criteria were reconstructed in three-dimensions (3D) with a 40×/1.4 N.A., Plan-Apochromat oil immersion objective on a Zeiss Axiophot 2 microscope equipped with a motorized stage, video camera system, and Neurolucida morphometry software (MBF Bioscience). Using NeuroExplorer software (MBF Bioscience) total dendritic length, number of intersections, and the amount of dendritic material per radial distance from the soma, in 30 μm increments were analyzed in order to assess morphological cellular diversity and potential differences among animals^45^.

### Confocal Laser Scanning Microscopy and Spine Acquisition

Using an approach that precludes sampling bias of spines, dendritic segments were selected with a systematic-random design^42^,^43^,^44^. Dendritic segments, 20–25 μm in length, on secondary and higher order branches and at 50 and 100 μm from the soma, were imaged on the Zeiss LSM 510 confocal microscope (Zeiss) using a 100×/1.4 N.A. Plan-Apochromat objective with a digital zoom of 3.5 and an Ar/Kr laser at an excitation wavelength of 488 nm. All confocal stacks were acquired at 512 × 512 pixel resolution with a z-step of 0.1 μm and approximately 1 μm above and below the identified dendritic segment, a pinhole setting of 1 Airy unit and optimal settings for gain and offset. On average 3 z-stacks were imaged per apical and basal tree and 5 neurons per animal. In order for a dendritic segment to be optimally imaged it had to satisfy the following criteria: (1) the entire segment had to fall within a depth of 50 μm; (2) dendritic segments had to be either parallel or at acute angles to the coronal surface of the section; and (3) segments did not overlap other segments that would obscure visualization of spines^42^,^43^,^44^. To improve voxel resolution and reduce optical aberration along the Z-axis, the acquired images were deconvolved using an interactive blind deconvolution algorithm (AutoDeblur version 8.0.2; MediaCybernetics).

### Spine Data Analysis

After deconvolution, the confocal stacks were analyzed using NeuronStudio software^46^,^47^,^48^ (http://www.mssm.edu/cnic) to examine global and local morphometric characteristics of dendritic spines, such as density, shape (stubby, mushroom, and thin), diameter and head volume. This software allows for automated digitization and reconstructions of 3D neuronal morphology from multiple confocal stacks on a spatial scale and averts the subjective errors encountered during manual tracing using a Rayburst-based spine analysis^46^,^47^,^48^. A spine was labelled thin or mushroom if the ratio of its maximum head diameter to maximum neck diameter was >1.1. Spines that fit those criteria and had a maximum head diameter of <0.35 μm were classified as thin spines, and otherwise they were classified as mushroom spines.

### Electron microscopy

Coronal sections (250 μm-thick) encompassing the CA1 region of the hippocampus were prepared for EM as reported previously^49^. Briefly, slices were cryoprotected in graded phosphate buffer/glycerol washes at 4 °C, and manually microdissected to obtain blocks containing the CA1 region. The blocks were rapidly freeze-plunged into liquid propane cooled by liquid nitrogen (−190 °C) in a Universal cryofixation System KF80 (Reichert-Jung) and subsequently immersed in 1.5% uranyl acetate dissolved in anhydrous methanol at −90 °C for 24 hours in a cryosubstitution unit (Leica). Block temperatures were raised from −90 to −45 °C in steps of 4 °C/hour, washed with anhydrous methanol, and infiltrated with Lowicryl resin (Electron Microscopy Sciences) at −45 °C. The resin was polymerized by exposure to ultraviolet light (360 nm) for 48 hours at −45 °C followed by 24 hours at 0 °C. Block faces were trimmed and ultrathin sections (90 nm) were cut with a diamond knife (Diatome) on an ultramicrotome (Reichert-Jung) and at least 5 serial sections were collected on formvar/carbon-coated nickel slot grids (Electron Microscopy Sciences).

### Quantitative analyses of synapse density

For synapse quantification, serial section micrographs were imaged at 15,000x on a Hitachi H-7000 transmission electron microscope using an AMT Advantage CCD camera (Advanced Microscopy Techniques). Nine sets of serial images across the same set of 5 consecutive ultrathin sections were taken from the SR of the hippocampal CA1 field and imported into Adobe Photoshop (version CS5, Adobe Systems). To obtain a stereologically unbiased population of synapses for quantitative morphologic analysis, we used a disector approach on ultrathin sections as in previous reports^43^,^50^,^51^. Briefly, all axospinous synapses were identified within the first and last 2 images of each 5-section serial set, and counted if they were contained in the reference image but not in the corresponding look-up image. To increase sampling efficiency, the reference image and look-up image were then reversed; thus each animal included in the current study contributed synapse density data from a total of 18 disector pairs. The total volume examined was 11.317 μm^3^, and the height of the disector was 180 nm. Axospinous synapse density (per μm^3^) was calculated as the total number of counted synapses from both images divided by the total volume of the disector. The criteria for inclusion as an axospinous synapse included the presence of a presynaptic terminal and a distinct PSD separated by a clear synaptic cleft. The synapse density was calculated as the total number of counted synapses divided by the total volume of the disectors used. For a synapse to be scored as perforated it had to display two or more separate PSD plates. Other ultrastructural synaptic parameters including PSD length, PSD area, and maximum spine HD were determined using a method previously described^43^,^51^. Briefly, all axospinous synapses in the third and fourth serial section were identified. Then, for each synapse, the longest PSD length and spine HD in those particular sections was identified and measured. For the smallest class of synapses that were only present in 1 serial section, measurements were taken in that section. For perforated synapses, the lengths of all PSD segments within a given section were summed and the total length was used in the statistical analyses.

### Western Blot

Mouse hippocampi were dissected from WT and K^b^D^b−/−^ mice (n = 4) and were immediately snap frozen and stored at −80 °C until processed. Hippocampi were homogenized in 2 mL of homogenization buffer (0.32 M sucrose, 1 mM EDTA, 5 mM HEPES, pH 7.4) using a dounce homogeniser and immediately mixed 3:5 with 2x Laemmli sample buffer. Homogenates were separated by SDS polyacylamide gel electrophoresis followed by transfer to a nitrocellulose membrane (Thermo Scientific, Rockford, USA) as described previously^52^. The primary antibodies used were 1:1,000 dilutions of mouse anti-PSD95 (Cat# 75–028, UC Davis/NIH NeuroMab Facility, Davis USA), mouse anti-GluN2B (Cat# 75–101, UC Davis/NIH NeuroMab Facility, Davis USA), goat anti-actin (Cat# sc-1615, Santa Cruz Biotechnology, Santa Cruz USA) and rabbit anti-GluA2/3 (Cat# AB1506, Millipore, Temecula USA) or 1:2:000 dilutions of rabbit anti-VGluT1 (Cat# ab180188, abcam, Cambridge USA) and rabbit anti-synaptophysin (Cat# ab32127, abcam). The secondary antibodies were 1:10,000 dilutions of AlexaFluor-680 conjugated donkey anti-mouse IgG H+L (Cat# A10038, Life Technologies, Eugene USA), AlexaFluor-680 conjugated donkey anti-rabbit (Cat# A10043, Life Technologies, Eugene USA) and AlexaFluor 790 conjugated donkey anti-goat IgG H+L (Cat# A11370, Life Technologies, Eugene USA). Fluorescence was detected and quantitated using Odyssey Infrared Imager (LI-COR, Lincoln USA). Signal intensities were normalized against β-actin. Results are expressed as fold change from the respective WT control and shown as mean ± SEM. All statistical analyses were carried out using Prism software (GraphPad Software).

### Statistical Analysis

For all the morphology studies, mean values from single cells were obtained and then averaged for each animal and were used for comparison of means. For the mean apical and basal dendritic length, individual unpaired two-tailed *t*-tests were performed comparing each group to the WT. Both Sholl analyses of distance and number of intersections were analyzed using repeated-measures ANOVA with Bonferroni’s *post-hoc* tests, with genotype as the between-group factor (all MHCI deficient models compared to WT) and distance from the soma as the repeated-measures factor^42^,^43^,^44^,^53^. Determination of changes in total dendritic spines as well as overall dendritic spine types were initially done by collapsing all spines at varying dendritic distances (overall analysis), and analyzed using a one-way ANOVA followed by Bonferroni’s *post-hoc* tests. Subsequent analyses were performed at specific dendritic distances from the soma (50 μm and 100 μm). Spine classification, volume, and head diameter were analyzed using a one-way ANOVA with Bonferroni’s *post-hoc* tests^38^,^39^,^40^,^41^,^42^,^43^,^44^,^53^. Synapse densities and Western blot analysis were analyzed using unpaired *t*-tests. The α level was set at 0.0125 for *t*-tests of neuronal morphology (to correct for multiple comparisons of 3 transgenic groups to WT) and 0.05 for all other statistical tests. All data were represented as mean ± SEM. All statistical analyses were carried out using Prism software (GraphPad Software).

## Additional Information

**How to cite this article**: Lazarczyk, M. J. *et al*. Major Histocompatibility Complex class I proteins are critical for maintaining neuronal structural complexity in the aging brain. *Sci. Rep*. **6**, 26199; doi: 10.1038/srep26199 (2016).

## Supplementary Material

Supplementary Information

## Figures and Tables

**Table 1:** Summary of the number of neurons, segments, and spines analyzed for each genotype.

Number of	WT	K^b^D^b−/−^	β2M^−/−^	TAP^−/−^
Animals	6	5	3	6
Cells	40	32	19	34
Segments (total)	310	254	159	281
Segments (apical)	155	126	78	141
Segments (basal)	155	128	81	140
Spines (total)	23,328	20,180	13,209	21,644
Spines (apical)	11,673	10,177	6,455	11,023
Spines (basal)	11,645	10,003	6,754	10,621
