# Supplementary material for: Major Histocompatibility Complex class I proteins are critical for maintaining neuronal structural complexity in the aging brain
Source: Sci Rep. 2016 May 27;6:26199. doi: 10.1038/srep26199 (PMC4882527; doi:10.1038/srep26199)
Supplement: Supplementary Information [file srep26199-s1.pdf]

MHC class I proteins are critical for maintaining neuronal structural complexity in the aging brain

Maciej J. Lazarczyk[a,b,c†], Julia E. Kemmler[d†], Brett A. Eyford[e], Jennifer A. Short[a,b], Merina Varghese[a,b], Allison Sowa[a,b], Daniel R. Dickstein[a,b], Frank J. Yuk[a,b], Rishi Puri[a,b] Kaan E. Biron[e,f], Marcel Leist[d], Wilfred A. Jefferies[e,f,g,h,i], Dara L. Dickstein[a,b*]

[a]Fishberg Department of Neuroscience, [b]Friedman Brain Institute, Icahn School of Medicine at Mount Sinai, New York, NY 10029, USA
[c]Department of Mental Health and Psychiatry, Division of General Psychiatry, University Hospitals of Geneva, Faculty of Medicine of the University of Geneva, Geneva, Switzerland
[d]University of Konstanz, Doerenkamp-Zbinden, Universitätsstrasse. 10, 78457 Konstanz, Germany
[e]Michael Smith Laboratories, The University of British Columbia, 2185 East Mall, Vancouver, British Columbia, V6T 1Z4, Canada;
[f]Department of Microbiology and Immunology, University of British Columbia, 1365 - 2350 Health Sciences Mall, Vancouver, BC, V6T 1Z3, Canada.
[g]Centre for Blood Research, University of British Columbia, 2350 Health Sciences Mall, Vancouver, BC, V6T 1Z3, Canada;
[h]Department of Zoology, University of British Columbia, 2370 - 6270 University Blvd., Vancouver, BC, V6T 1Z4, Canada;
[i]Department of Medical Genetics, 1364 - 2350 Health Sciences Mall, Vancouver, BC, V6T 1Z3, Canada

† These authors contributed equally to this work: Maciej J. Lazarczyk and Julia E. Kemmler

* Corresponding author:
Dara L. Dickstein Ph.D.,
Fishberg Department of Neuroscience, Icahn School of Medicine at Mount Sinai, One Gustave L. Levy Place, Box 1639, New York, NY 10029 USA
(Ph) 212-824-9304 (fx) 646-537-9585
dara.dickstein@mssm.edu.

Supplementary Fig. 1

NMDA receptor expression is increased in MHCI deficient mice. Full length Western blots of representative blots depicted in Fig. 8. Hippocampal homogenates were analyzed for PSD95, GluA2/3, and GluN2B levels in (a) 3 month and (b) 12 month old $K^bD^{b-/-}$ and WT mice. All proteins were normalized to actin.

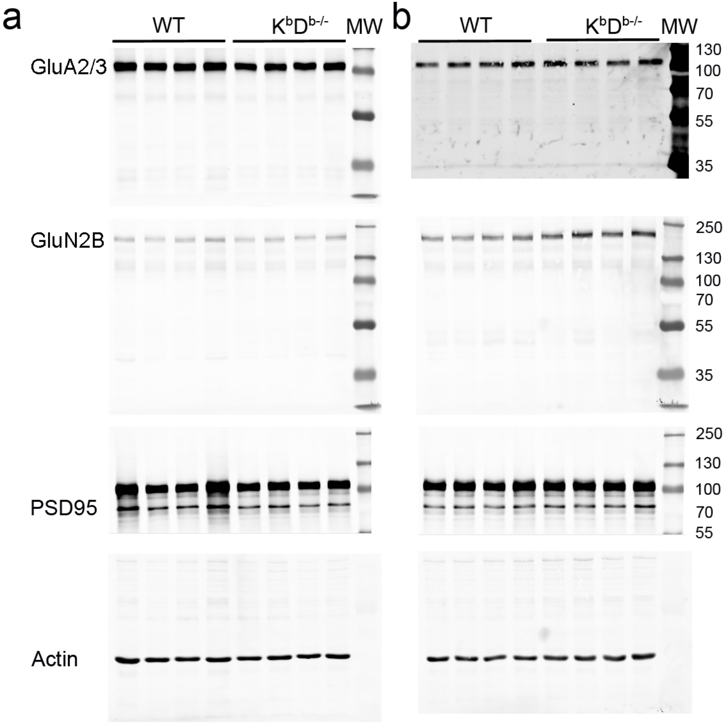

a
b
WT
K^{b}D$^{b-/-}$
MW
GluA2/3
GluN2B
PSD95
Actin
130
100
70
55
35
250
130
100
70
55
35
250
130
100
70
55

Supplementary Fig. 2

Presynaptic markers are unchanged in MHCI deficient mice. Full length Western blots of representative blots depicted in Fig. 9. Hippocampal homogenates were analyzed for vGluT1 and synaptophysin in (a) 3 month and (b) 12 month old $K^bD^{b-/-}$ and WT mice. All proteins were normalized to actin.

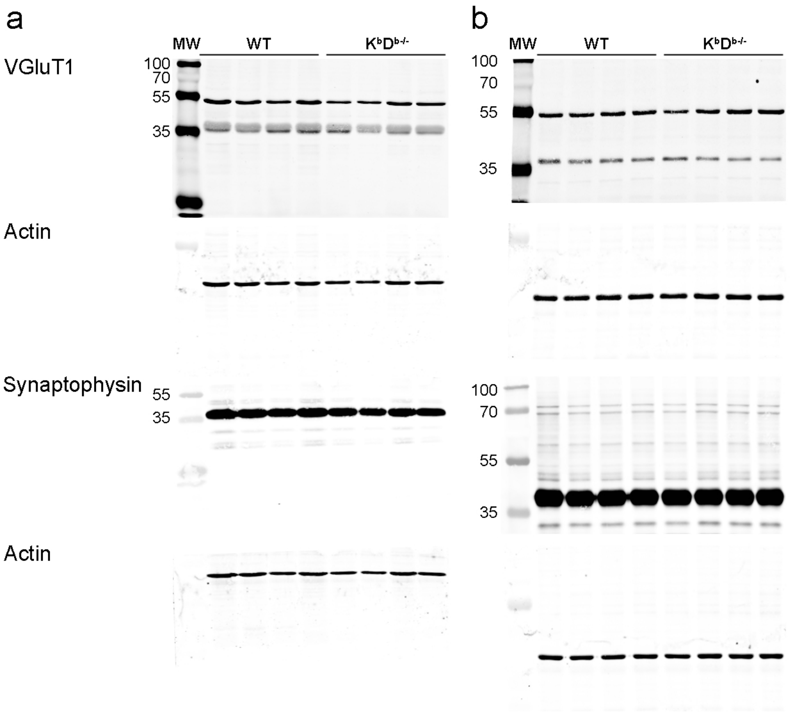
a
b
MW
WT
K^bD^b-/-
VGluT1
100
70
55
35
Actin
Synaptophysin
55
35
Actin
MW
WT
K^bD^b-/-
100
70
55
35
100
70
55
35
